# Direct protein introduction into plant cells using a multi-gas plasma jet

**DOI:** 10.1371/journal.pone.0171942

**Published:** 2017-02-09

**Authors:** Yuki Yanagawa, Hiroaki Kawano, Tomohiro Kobayashi, Hidekazu Miyahara, Akitoshi Okino, Ichiro Mitsuhara

**Affiliations:** 1 Institute of Agrobiological Sciences, NARO, Kannondai, Tsukuba, Ibaraki, Japan; 2 FIRST, Institute of Innovative Research, Tokyo Institute of Technology, Nagatsuta, Midori-ku, Yokohama, Japan; University of Tsukuba, JAPAN

## Abstract

Protein introduction into cells is more difficult in plants than in mammalian cells, although it was reported that protein introduction was successful in shoot apical meristem and leaves only together with a cell-penetrating peptide. In this study, we tried to introduce superfolder green fluorescent protein (sGFP)-fused to adenylate cyclase as a reporter protein without a cell-penetrating peptide into the cells of tobacco leaves by treatment with atmospheric non-thermal plasmas. For this purpose, CO_2_ or N_2_ plasma was generated using a multi-gas plasma jet. Confocal microscopy indicated that sGFP signals were observed inside of leaf cells after treatment with CO_2_ or N_2_ plasma without substantial damage. In addition, the amount of cyclic adenosine monophosphate (cAMP) formed by the catalytic enzyme adenylate cyclase, which requires cellular calmodulin for its activity, was significantly increased in leaves treated with CO_2_ or N_2_ plasma, also indicating the introduction of sGFP-fused adenylate cyclase into the cells. These results suggested that treatment with CO_2_ or N_2_ plasma could be a useful technique for protein introduction into plant tissues.

## Introduction

Protein introduction into organic cells is a valuable technique for not only basic research but also for industrial uses. In mammals, protein introduction techniques such as transfection using cell-penetrating factors dependent on endocytosis and bacterial delivery have already been used, and various protein transfection reagents are available commercially [1–3]. Thus, there have been numerous reports using protein introduction techniques such as genome editing and functional inhibition of proteins in mammalian systems [2–4]. However, in plants, the transfection systems used in mammalian cells do not work well, which is probably because of the different structure of the cell surface and cell wall. A previous report showed that florigen, which is a small protein that promotes flowering, was introduced into shoot apical meristems by soaking with cell-penetrating peptides (CPPs) [5]. In addition, proteins fused to CPPs were introduced into leaves by infiltration, which involves injecting a solution into the intercellular spaces using a syringe [6]. However, the techniques using CPPs still have limitations in depending of the tissues and/or methods involved. Thus, novel techniques for protein introduction into plant cells are needed in order expected to be developed to advance plant research.

Non-thermal atmospheric-pressure plasmas have attracted attention in various fields such as the manufacturing and pharmaceutical industries and in environmental control [7–8]. Previously, we reported that a multi-gas plasma jet was able to cause surface hydrophilization of polyimide films [9]. It was also reported that anesthetic gas and toxic chemicals could be decomposed using atmospheric-pressure plasmas [10, 11]. Regarding the effects on organisms, non-thermal atmospheric-pressure plasmas inactivated bacteria and biomolecules [12–16]. Because the plasma damaged the cell surface of bacteria, it may act on the cell surface of plant tissues as well. Indeed, the surface structure of coriander seeds was partially destroyed with a roughening of the surface by treatment with direct barrier discharge plasma [17]. In mammalian cells, plasma treatment promoted GFP introduction into the cells together with a CPP [18]. These findings imply the possibility that plasma treatment could disturb the surface structure of plant cells leading to the introduction of organic matter, such as proteins, into the cells, although plant cells have cell walls in addition to a cell membrane.

In this study, we founded that treatment with CO_2_ or N_2_ plasma was effective for protein introduction into plant cells without the use of any CPP. This method will be expected to be helpful for various aspects of research and industrial uses such as genome editing, functional inhibition, and flowering control in plants.

## Materials and methods

### Plant material, plant medium, and antibody

Tobacco (*Nicotiana tabacum* cv. Samsun NN) plants were grown in a growth room at 25°C in a 16 /8 h light/dark cycle. Half-strength Murashige and Skoog (MS) salt medium was used to cultivate leaf pieces. Seeds of *Arabidopsis thaliana* (ecotype Columbia-0; Col-0) were sown on soil and were grown in a growth chamber at 22°C in a 12 /12 h light/dark cycle. Rice (*Oryza sativa* cv Nipponbare) plants were grown using a tap water in a growth chamber at 27°C in a 16 /8 h light/dark cycle. An anti-GFP antibody was purchased from Abcam (Tokyo, Japan).

### Preparation of sGFP-fused adenylate cyclase (CyaA) and sGFP-fused CyaA-R8 proteins

Information on all primers used in this study is given in S1 Table.

For the preparation of His-tagged sGFP-CyaA protein, the sGFP DNA fragment was amplified using primers BamHI-sGFP-F and EcoRI-sGFP-R. The resulting fragment was digested with *Bam*HI and *Eco*RI and inserted into a pENTR3C vector (Invitrogen) to produce pENTR-sGFP. An open-reading frame (ORF) encoding 400 N-terminal amino acids of CyaA was amplified using primers EcoRI-Cya-F and XhoI-stop-Cya1200R. The resulting fragment was digested with *Eco*RI and *Xho*I and inserted into pENTR3C-sGFP. The sGFP-CyaA fragment was isolated by digesting with *Bam*HI and *Xho*I, and this was inserted into a pET28a vector (Novagen) carrying a 6 × histidine (His) tag at the N-terminus. For the preparation of His-tagged sGFP-CyaA-R8 protein, a DNA fragment encoding 8 arginine amino acids (R8) was produced by annealing two single strand DNAs EcoRI-R8-stop-XhoI-F and XhoI-stop-R8-EcoRI-R and was then inserted into pENTR3C-sGFP-CyaA digested with *Eco*RI and *Xho*I. An ORF encoding the 400 N-terminal amino acids of CyaA was amplified using primers EcoRI-Cya-F and EcoRI-Cya1200R. The resulting fragment was digested with *Eco*RI and inserted into pENTR-sGFP-R8. The sGFP-CyaA-R8 fragment was digested with *Bam*HI and *Xho*I and inserted into a pET28a vector.

The resulting plasmids pET28a-sGFP-CyaA and pET28s-sGFP-CyaA-R8 were used to transform BL21 (DE3) cells. Expression and purification of His-tagged sGFP-CyaA and His-tagged sGFP-CyaA-R8 were performed using Ni sepharose^TM^ high performance in accordance with manufacturer’s instructions (GE healthcare, Tokyo, Japan).

### Plasma treatment

**Fig 1:**
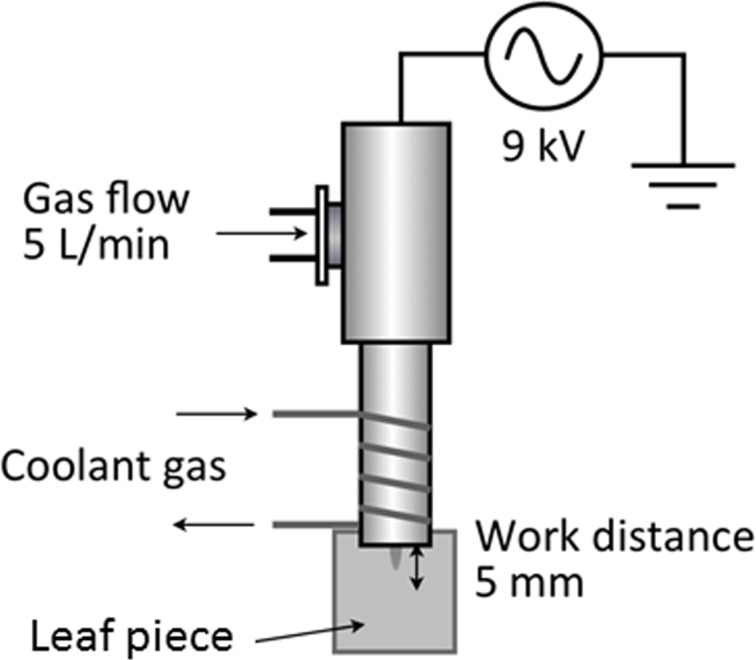
Schematic of plasma treatment. The outlet for the plasma was placed immediately above the leaf piece. The distance between the outlet and the leaf piece was 5 mm.

Recently, plasma jet sources that can generate non-thermal plasma have been developed and used to apply plasma without heat and electric damages in heat-sensitive objects such as living bodies or cells [19–27]. Plasma is expelled from an aperture in the plasma source using a gas flow. The present experiments were conducted using a multi-gas plasma jet source developed by our group [9]. The body of the device was grounded and the internal high-voltage electrode was connected to an AC power supply (Plasma Concept Tokyo Inc.) of 16 kHz and 9 kV at approximately 10 W (Fig 1). Stable atmospheric-pressure plasmas composed of various gas species, such as argon (Ar), oxygen (O_2_), nitrogen (N_2_), carbon dioxide (CO_2_), mock air (mixture of 20% O_2_ and 80% N_2_), and a mixture of H_2_ and Ar (5% H_2_ and 95% Ar), were generated through a 1 mm aperture with a flow rate of 5 L/min. The temperature of the generated plasmas was below 50°C at 5 mm from the outlet using thermocouple measurement. To generate lower temperature plasma (approximately 20–30°C), the gas was cooled using a gas-cooling device that uses liquid nitrogen, as described previously [28]. Tobacco leaves were cut into squares of approximately 1.5–2 cm along each side. After plasma treatment of the leaf piece, it was placed in a well of 12-well culture plate with 400 μL/well of phosphate-buffered saline (PBS) with or without 50 μg/mL His-sGFP-CyaA-R8 (265 pmol) or His- sGFP-CyaA (271 pmol). Rosette leaves detached from Arabidopsis plants and rice roots cut into 1–2 cm lengths were treated by plasmas and then placed in a well of 12-well culture plate with 400 μL/well of protein solution as similar to tobacco leaves.

### cAMP enzyme immunoassay

For quantitative analysis, the amount of cAMP was measured using an Amersham cAMP Biotrak Enzymeimmunoassay System in accordance with the manufacturer’s instructions (GE). Leaf discs of 13 mm diameter were prepared using a cork borer and ground in a mortar and a pestle with liquid nitrogen, and the powder was further ground in 320 μL of 6% (w/v) trichloroacetic acid. Two-hundred microliters of the homogenate were centrifuged at 2,000 g for 15 min at 4°C. The supernatant was washed with 5 volumes of water saturated diethyl ether 4 times. The remaining aqueous extract was dried in a vacuum drier at 55°C. The dried extract was dissolved in 200 μL of assay buffer supplied with the kit. Forty microliters of each dissolved extract was used for the cAMP enzyme immunoassay.

### Confocal microscopy

GFP, intrinsic fluorescence, and bright field images were captured using a confocal laser scanning microscope FV-300 (Olympus Corp.) with Fluoview software (Olympus Corp.).

## Results

### Selection of effective gas sources for a lower temperature multi-gas plasma jet for protein introduction into the cells of tobacco leaves

**Fig 2:**
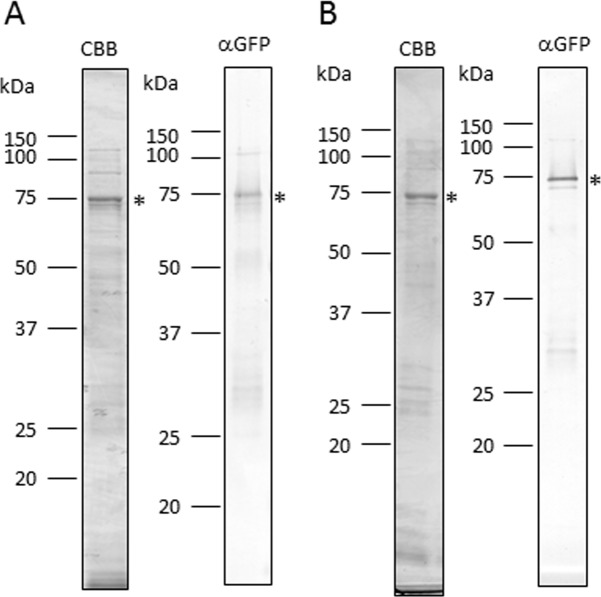
**Quality check of purified His-sGFP-CyaA-R8 (A) and His-sGFP-CyaA (B).** Coomassie brilliant blue staining or immunoblotting using an anti-GFP antibody (αGFP) was performed using 1 μg or 50 ng of purified proteins, respectively. Asterisks indicate protein bands that correspond to purified His-sGPF-CyaA-R8 and His-sGFP-CyaA.

**Fig 3:**
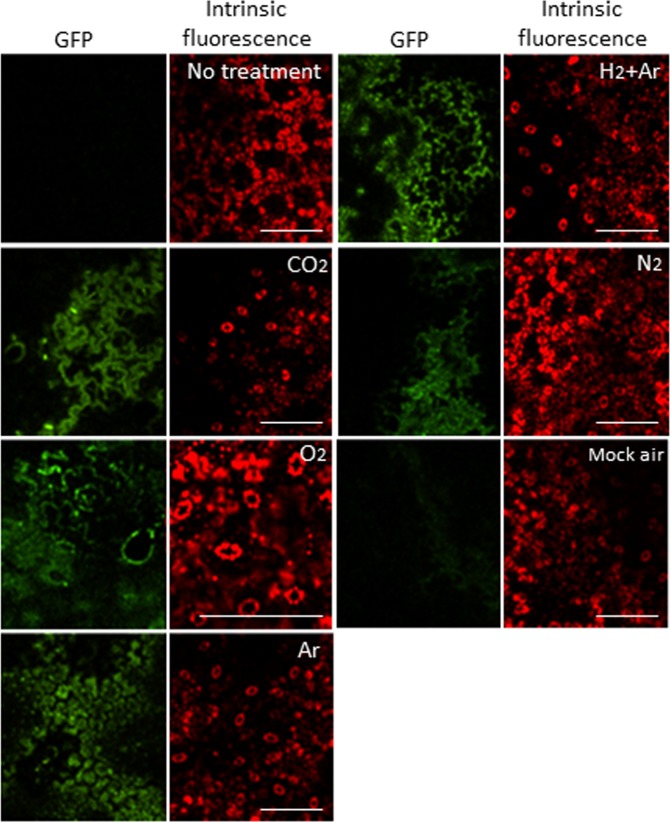
GFP fluorescence was examined by a confocal microscopy to select effective gas sources for protein introduction. PBS with His-sGFP-CyaA-R8 was applied onto leaf pieces of tobacco after plasma treatment using CO_2_, O_2_, Ar, a mixture of H_2_ and Ar, N_2_ or mock air gas plasma treatment for 30 s. After leaf pieces were kept in the solution for 1 day, GFP (green) and intrinsic fluorescence (red) images were captured using a confocal microscope. GFP and intrinsic fluorescence show the subcellular localization of His-sGFP-CyaA-R8 and chloroplasts, respectively. No plasma treatment was used as a negative control. Bar; 200 μm.

**Fig 4:**
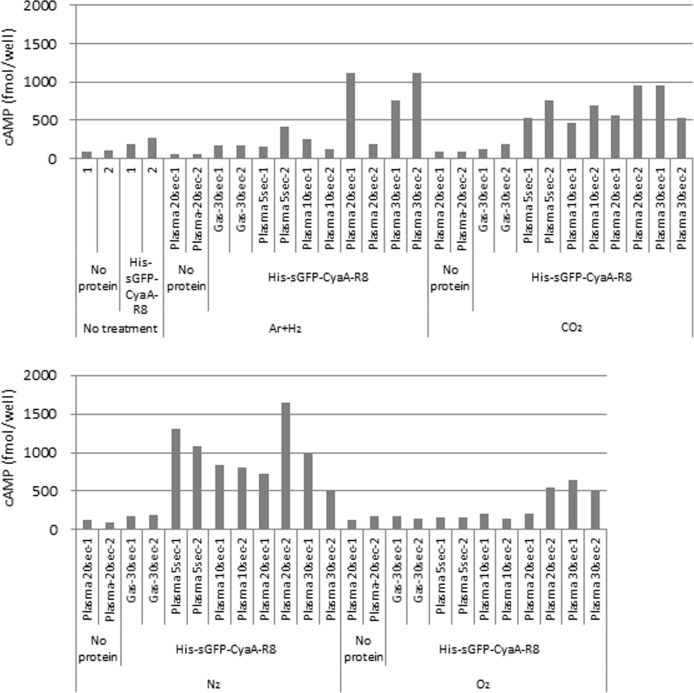
Amount of cAMP was measured in leaf pieces of tobacco to further narrow down suitable gas sources for protein introduction. PBS with or without His-sGFP-CyaA-R8 was applied onto leaf pieces after plasma treatment with a mixture of Ar and H_2_, CO_2_, N_2_, or O_2_ gases for 5 (Plasma-5sec), 10 (Plasma-10sec), 20 (Plasma-20sec), or 30 s (Plasma-30sec). After leaf pieces were kept in the solution for 1 day, assay samples were prepared from leaf discs produced as described in the Materials and Methods. Forty microliters of each prepared sample as described in the Materials and Methods was applied into a 96-well plate of the assay kit. The amount of cAMP was calculated per well using a standard curve made with non-acetylation standard cAMP from the assay kit. A mixture of Ar and H_2_, CO_2_, N_2_, or O_2_ gas was used as a negative control against each plasma (Gas-30sec). No treatment was used as a negative control against plasma treatment. No protein was used as a negative control against protein treatment. Two independent samples were tested for narrow down suitable gas sources for further detail experiments. Number “-1” and “-2” indicate samples 1 and 2, respectively.

It is known that the application of CPPs enables the delivery of proteins into plant cells [5, 6]. Thus, to select suitable gas sources for protein introduction by plasma treatment, a solution with purified CPP R8 (RRRRRRRR)-fused sGFP-CyaA (His-sGFP-CyaA-R8) (Fig 2A) was applied to leaf pieces that had been treated with various gas plasmas controlled at approximately 20–30°C (lower temperature plasma). In general, temperature of non-thermal atmospheric-pressure plasmas is approximately 40–50°C. However, most plant species grow at lower temperatures than those generated by non-thermal atmospheric-pressure plasmas. Indeed, tobacco plants grow at 25°C, and temperatures over 40°C is too high for their survival. Previously, our group developed a temperature-controllable multi-gas plasma jet source generating stable atmospheric-pressure plasma using various gas sources [9, 28]. Thus, we used this system to make lower temperature plasma for our experiments. Leaf pieces were treated with plasma using a mixture of Ar and H_2_, CO_2_, N_2_, or O_2_ as a gas source, followed by incubation with a solution of CPP R8 (RRRRRRRR)-fused sGFP-CyaA. As shown in Fig 3, GFP fluorescence was observed inside the cells after plasma treatment using gas sources with the mixture of Ar and H_2_, CO_2_, N_2_, and O_2_. No fluorescence was observed in cells treated with mock air plasma. GFP fluorescence was observed in leaves treated with Ar plasma, but the fluorescence was unclear in its shape, implying that many proteins were still outside of the cells. Thus, we considered that the mixture of Ar and H_2_, CO_2_, N_2_, and O_2_ were more suitable as gas sources of plasma production than those with mock air or Ar for protein introduction into tobacco cells. Next, to further narrow down efficient gas sources, a quantitative analysis of cAMP in leaves was performed as a further evaluation method (Fig 4). CyaA catalyzes cAMP formation dependent on the presence of calmodulin protein in the cytoplasm and ATP in living cells. Thus, the amount of cAMP represents the amount of CyaA protein introduced into living cells. In Fig 4, the amount of cAMP in the leaf was measured after treatment with plasma produced using gas sources of the mixture of Ar and H_2_, CO_2_, N_2_, and O_2_. cAMP amounts were increased by treatment with plasmas generated using CO_2_ and N_2_ gas sources in samples treated for 5, 10, 20, and 30 s compared with their gas treatment. cAMP amounts were increased in samples treated with plasmas generated using the mixture of Ar and H_2,_ and O_2_ gas sources with 20 and 30 s treatment, but not after 5 or 10 s treatment. The result showed that CO_2_ and N_2_ plasmas could introduce sGFP-CyaA-R8 more quickly than plasmas generated using the mixture of Ar and H_2,_ and O_2_ gas sources. Shorter plasma treatment is probably more efficient than longer plasma treatment in terms of quicker treatment, cheaper gas cost, and less damage to the tissues. Thus, we decided to use CO_2_ and N_2_ plasmas for further experiments.

**Fig 5:**
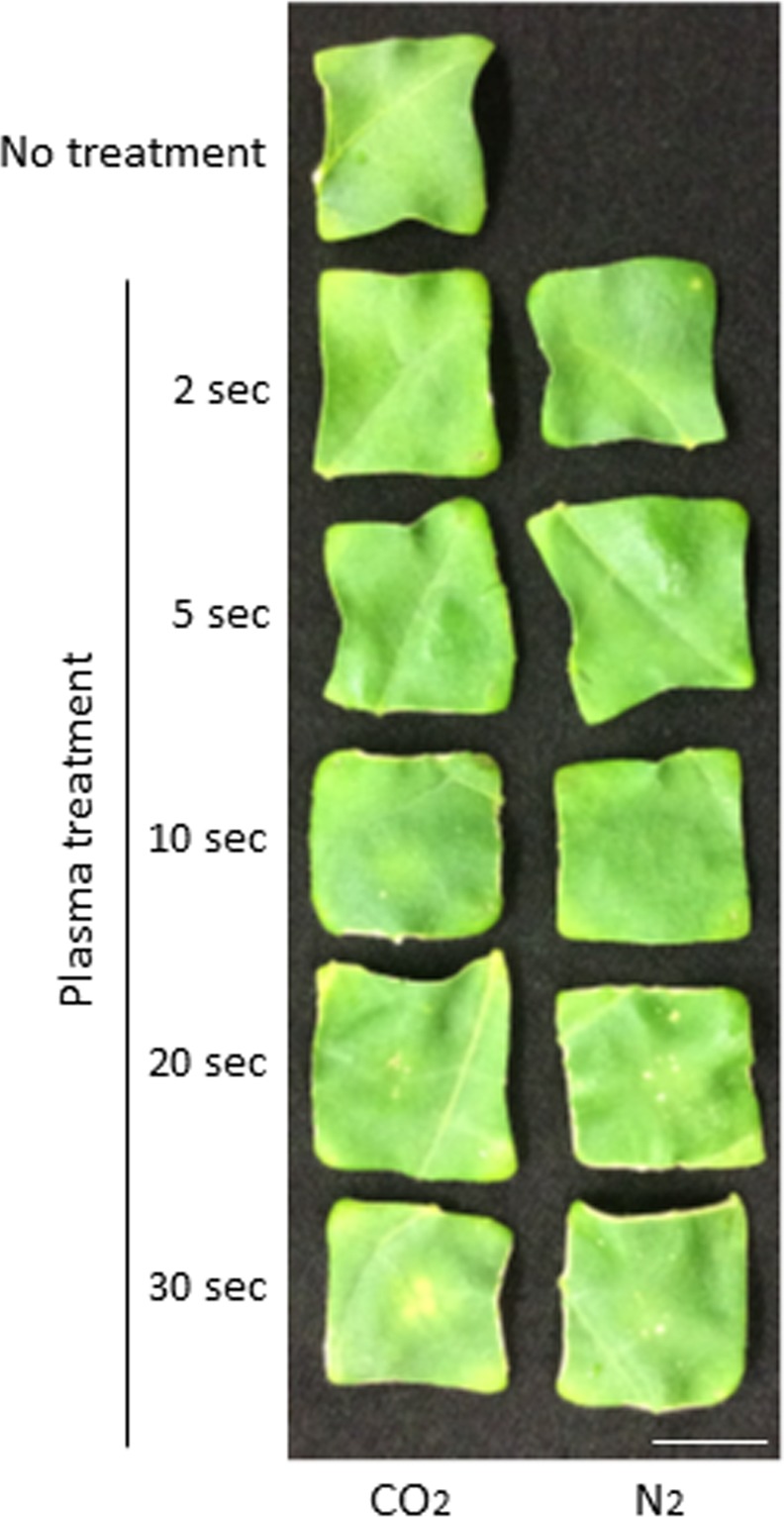
Lower-temperature plasma caused no significant damage to leaf pieces of tobacco. After CO_2_ or N_2_ plasma treatment for 2, 5, 10, 20, or 30 s, leaf pieces were cultured on half-strength MS media plates for 6 days. A leaf piece without any treatment was used as a negative control. Bar; 1 cm.

**Fig 6:**
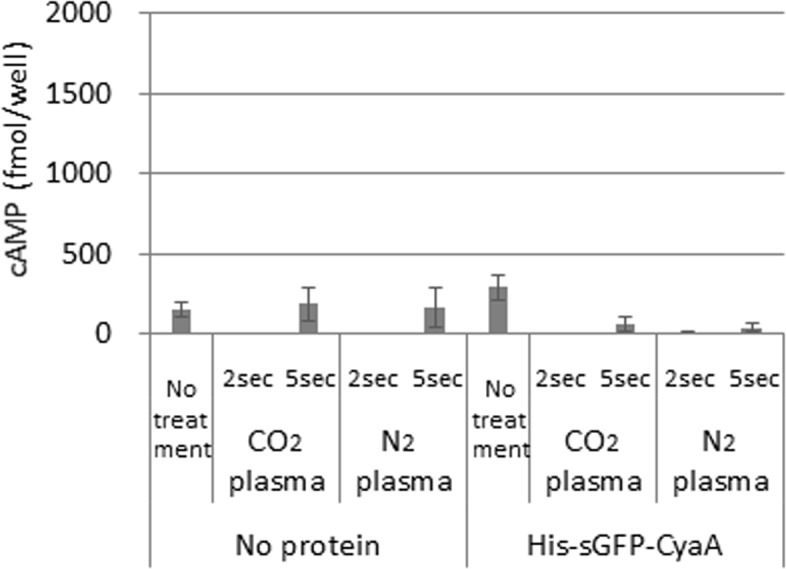
Amount of cAMP was measured after plasma treatment without temperature control. PBS with His-sGFP-CyaA or no protein (No protein) was applied onto leaf pieces of tobacco after plasma treatment using CO_2_ or N_2_ gas. No treatment was used as negative control. After leaf pieces were kept in the solution for 1 day, assay samples were prepared from leaf discs produced as described in the Materials and Methods. Forty microliters of each prepared sample as described in the Materials and Methods was applied into a 96-well plate of the assay kit. The amount of cAMP was calculated per well using a standard curve made with non-acetylation standard cAMP from the assay kit. Error bars indicate standard deviation from 6 independent biological replicates.

CO_2_ and N_2_ plasmas have the ability to inactivate several microorganisms [13], implying that the plasmas should affect cell survival in multicellular organisms as well. Thus, we examined whether our treatment method with CO_2_ and N_2_ plasmas may damage plant tissues. As shown in Fig 5, no significant damage was observed in leaf pieces kept for 6 days after CO_2_ plasma treatment for 2 or 5 s and N_2_ plasma treatment for 2, 5 or 10 s. Damage to the leaf pieces was observed when they were treated with CO_2_ plasma for 10, 20 or 30 s and N_2_ plasma for 20 or 30s in part. To confirm the importance of lower temperature, we examined whether cAMP levels were increased by plasmas generated by non-temperature controlled CO_2_ or N_2_ gas source with a higher temperature of approximately 40–50°C. Every experimented samples showed low level of cAMP amount, which does not exceed no plasma treatment (No treatment) nor no protein as negative controls, indicating that His-sGFP-CyaA protein was not introduced into plant cells by treatment with higher-temperature plasma (Fig 6).

Considering these results, lower temperature plasmas generated using CO_2_ and N_2_ gas sources could be effective for introducing proteins into plant cells. Thus, lower temperature CO_2_ and N_2_ plasmas were used for further experiments.

### Protein introduction into cells of tobacco leaves, Arabidopsis leaves and rice roots without any CPP using CO_2_ and N_2_ plasmas

**Fig 7:**
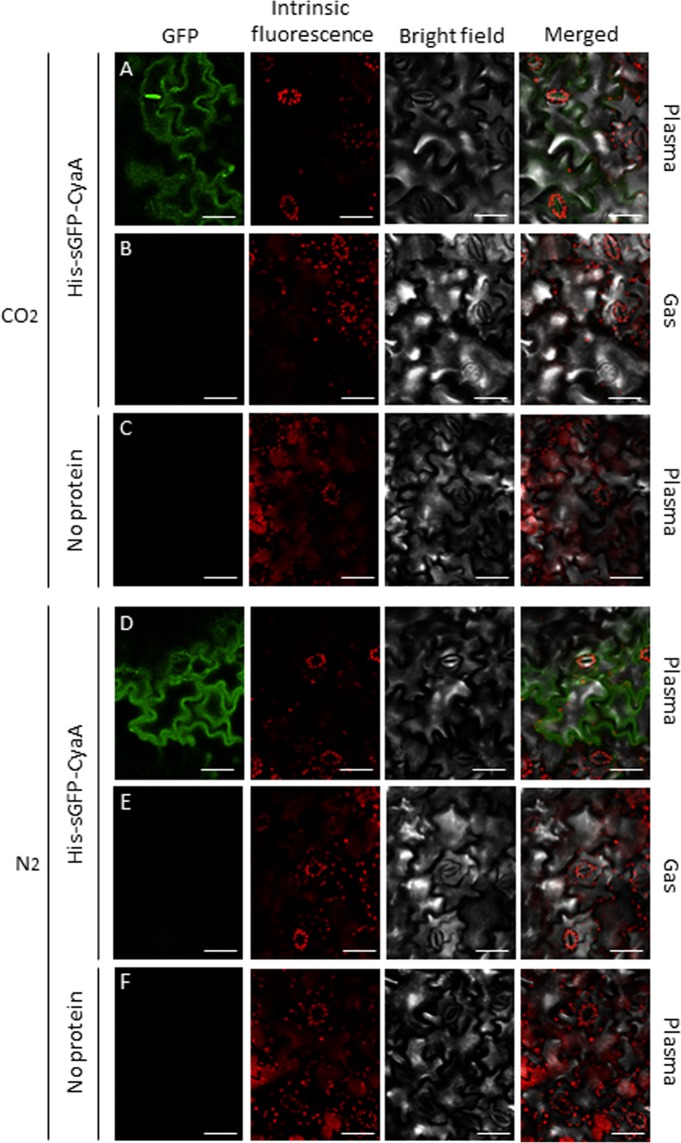
Subcellular localization of introduced His-sGFP-CyaA in cells of tobacco leaves. Leaf pieces were treated with PBS with His-sGFP-CyaA after CO_2_ (A) or N_2_ (D) plasma treatment. Leaf pieces were treated with PBS with His-sGFP-CyaA after CO_2_ (B) or N_2_ (E) gas treatment as a negative control. Leaf pieces were treated with PBS without any protein after CO_2_ (C) or N_2_ (F) plasma treatment. After leaf pieces were kept in their solutions for 1 day, GFP (green), intrinsic fluorescence (red), and bright field images were captured using a confocal microscope. GFP and intrinsic fluorescence show the subcellular localization of His-sGFP-CyaA and chloroplasts, respectively. Plasma treatment was performed for 5 s in all experiments. Bars; 50 μm.

**Fig 8:**
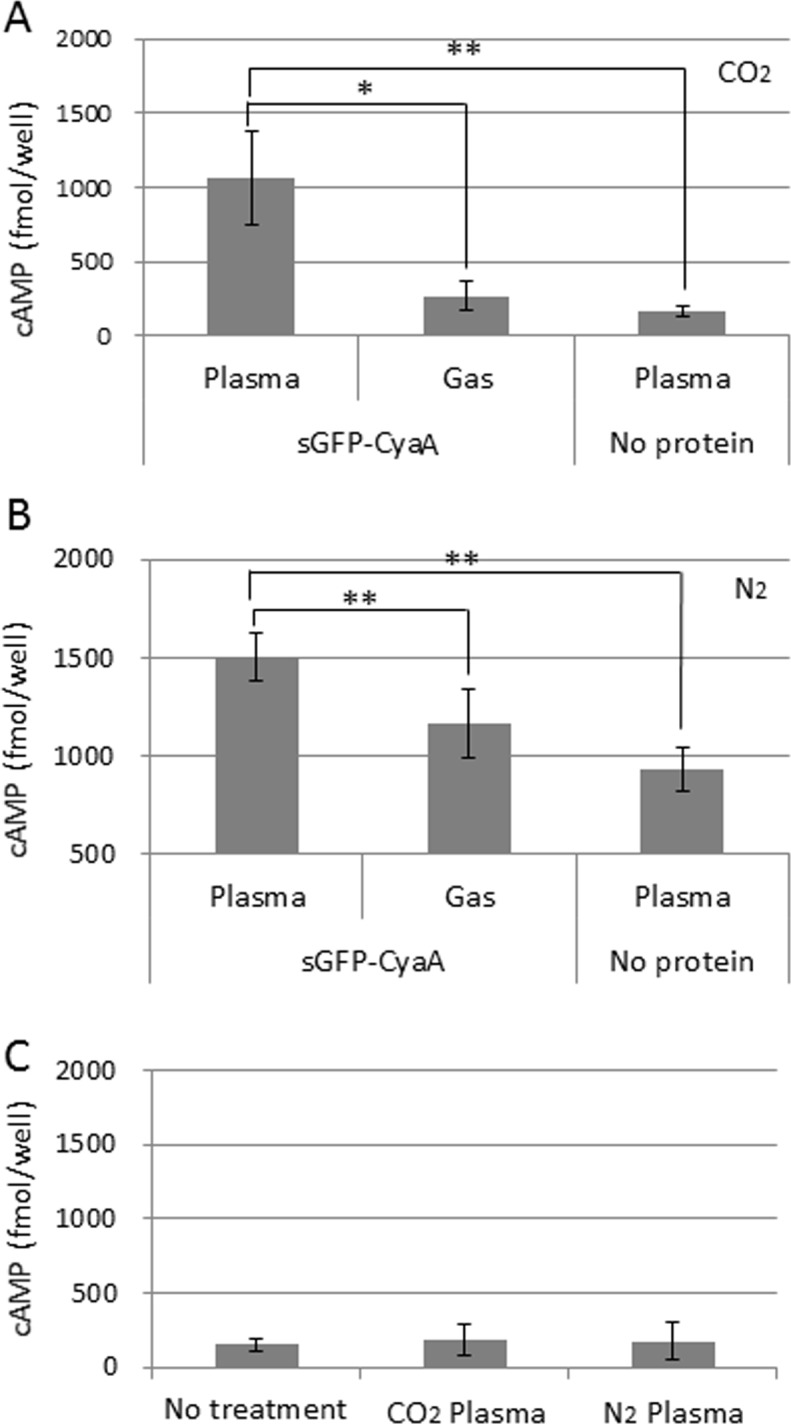
Quantitative analysis of His-sGFP-CyaA introduced into leaf pieces of tobacco. The amount of cAMP was measured after treatment of CO_2_ or N_2_ plasma or gas for 5 s. The effect of CO_2_ (A) or N_2_ (B) plasma for the introduction of His-sGFP-CyaA into leaf pieces was examined. No protein was applied onto leaf pieces after plasma treatment or with no treatment (C). After leaf pieces were kept in the solution for 1 day, assay samples were prepared from leaf discs produced as described in the Materials and Methods. Forty microliters of each prepared sample as described in the Materials and Methods was applied into a 96-well plate of the assay kit. The amount of cAMP was calculated per well using a standard curve made with non-acetylation standard cAMP from the assay kit. Error bars indicate standard deviation from 3 independent biological replicates. Asterisks indicate significant differences analyzed using Student’s t-test compared with the relevant vector control at p<0.05(*) or p<0.01(**).

To examine whether a CPP is required for protein introduction into plant cells using plasma treatment, we generated His-sGFP-CyaA protein without a CPP peptide, and the purified His-sGFP-CyaA (Fig 2) was applied onto leaf pieces after treatment with CO_2_ and N_2_ plasmas. As shown in Fig 7A and 7B, GFP fluorescence was observed in the cells after treatment with CO_2_ and N_2_ plasmas. As a negative control, His-sGFP-CyaA was applied onto leaf pieces treated with CO_2_ or N_2_ gas. As expected, no significant GFP signal was detected in either the cells or intracellular spaces after treatment with CO_2_ or N_2_ gas (Fig 7C and 7D). To obtain quantitative evidence of protein introduction into the plant cells, the amount of cAMP was measured (Fig 8). As expected, the amount of cAMP was significantly increased after treatment with CO_2_ (approximately 4.0 times) or N_2_ (approximately 1.3 times) plasma compared with CO_2_ or N_2_ gas. Next, to confirm whether the increase in cAMP was caused by His-sGFP-CyaA introduction into the cells by plasma treatment, the amount of cAMP was measured in leaf pieces with no protein exposure after plasma treatment. As expected, there was no significant difference between leaf samples with and without plasma treatment (Fig 8). In addition, no green fluorescence was observed in leaves without plasma treatment, whereas intrinsic fluorescence of the chloroplasts was still observed (Fig 7E and 7F). These results indicated that a CPP was not necessary for protein introduction into tobacco leaves.

**Fig 9:**
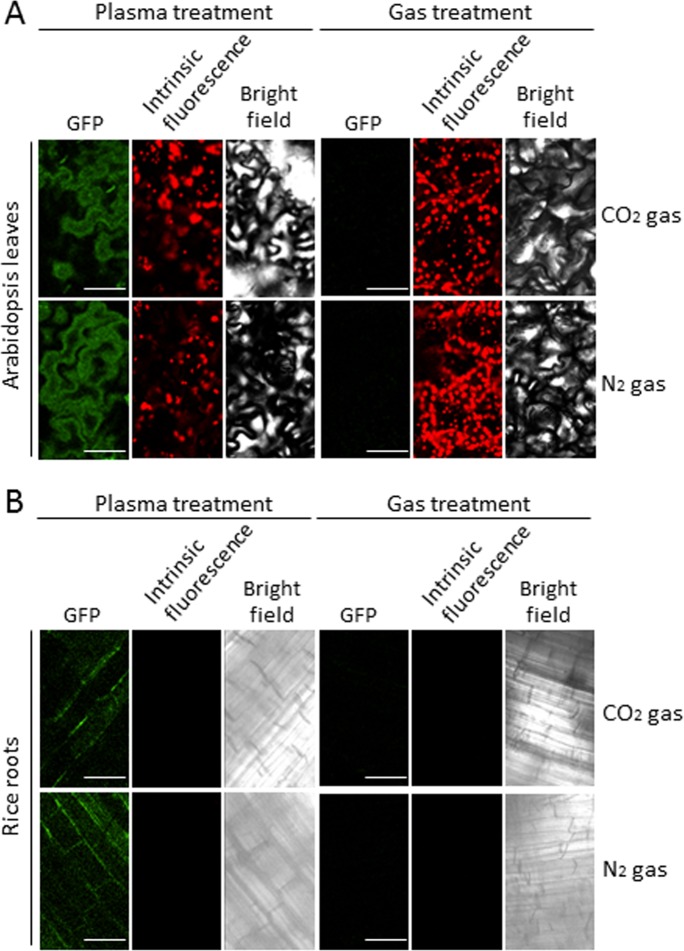
**Subcellular localization of introduced His-sGFP-CyaA in the cells of Arabidopsis leaves (A) and rice roots (B).** Arabidopsis leaves or rice roots were treated with PBS with His-sGFP-CyaA after CO_2_ (A) or N_2_ (D) plasma treatment for 2 s. CO_2_ or N_2_ gas was used as a negative control. After the leaves and roots were kept in the solutions for 1 day, GFP (green), intrinsic fluorescence (red), and bright field images were captured using a confocal microscope. GFP and intrinsic fluorescence show the subcellular localization of His-sGFP-CyaA and chloroplasts, respectively. Note that roots do not have any chloroplasts implying no intrinsic fluorescence. Bars; 50 μm.

Next, we examined whether CO_2_ or N_2_ plasma works for introducing proteins into cells of other plant species. For this purpose, purified His-sGFP-CyaA was applied onto Arabidopsis leaves and rice roots after treatment with CO_2_ or N_2_ plasma (Fig 9). As expected, CO_2_ and N_2_ plasmas were able to introduce His-sGFP-CyaA into both cells of Arabidopsis leaves and rice roots.

## Discussion

In this study, we demonstrated that His-sGFP-CyaA protein was introduced into cells of tobacco leaves (Figs 7 and 8), which have cell walls in addition to cell membranes using temperature controlled non-thermal atmospheric plasma generated with CO_2_ or N_2_ gas source. In addition to tobacco leaves, His-sGFP-CyaA was introduced into Arabidopsis leaves and rice roots by treatment with temperature controlled CO_2_ or N_2_ plasma (Fig 9). Thus, this technique can be applied to these plants. Non-temperature controlled plasmas with a higher temperature of approximately 40–50°C did not work for protein introduction into tobacco leaves (Fig 6). Thus, temperature control of plasmas to approximately 20–30°C must be important for protein introduction into these plant cells. So far there have been no reports of protein introduction into plant tissues without the assistance of a CPP. In addition, organic matter, such as proteins and nucleic acids, needs to contact directly with the cells via protoplasts or infiltration in order for them to be introduced into plant cells in many cases [29–31]. Plasma treatment was shown not to require a CPP in Figs 7, 8 and 9, although a CPP-fused protein was also introduced by plasma treatment (Fig 3), indicating the presence of a CPP did not affect protein introduction. Moreover, plasma treatment does not need infiltration, or the use of protoplasts to introduce proteins into tobacco, Arabidopsis and rice tissues from intact plant organs. These results suggested that proteins could pass through the surface cuticle, cell wall and cell membrane into the cells. Moreover, multi-gas plasma jet apparatus can be modified in size as needed. Thus, our technique using plasma could contribute to the introduction of proteins into plant tissues by means of a simple system that can be applicable for large-scale systems.

It was reported that CO_2_ and N_2_ plasmas produced higher amounts of reactive species such as OH and singlet oxygen radicals than plasmas generated using other gas sources [32]. It is thought that the effects of plasma treatment are contributed to by chemically reactive species such as OH and singlet oxygen radicals [13]. This implies that the higher amounts of reactive species produced by CO_2_ and N_2_ plasmas affect membrane integrity leading to protein introduction into tissues. Because plasma generated by other gas sources also produces OH and singlet oxygen radicals, these plasmas may introduce proteins into tobacco tissues by optimizing the method of plasma treatment.

General CPPs have positive charges, and charge is known to be an important factor for the introduction of organic matter by endocytosis [29–31]. Nevertheless, our results indicated that no CPP was necessary for protein introduction into tobacco, Arabidopsis and rice cells using plasma treatment. Electroporation is a widely used technique to introduce DNA and RNA into the cells by making pores electrically in the cell membranes. Moreover, particle guns introduce DNA and RNA together with metal particles by making pores. These observations indicate that making small pores in the cell surface is useful for introducing organic matter. Using a scanning electron microscope, it was reported that plasma generated by air gas sources damaged the cell surface of bacteria [14]. In plants, treatment with N_2_ plasma caused a roughening of the surface of coriander seeds [17]. Thus, proteins may be introduced into cells through pores made by plasma treatment of the cell wall and cell membrane of tobacco leaves. CO_2_ or N_2_ plasma treatment for at least 2–5 s did not affect the survival of tobacco leaves; however, longer plasma treatment damaged leaf cells in this experiments (Fig 5). These results imply that plasma treatment using our method may be sufficiently strong to make pores for protein introduction, but not so strong as to affect cell survival of tobacco leaves. Macromolecules and particles can also be transferred into living cells by structural changes in the cell membrane called endocytosis. It is known that plasma treatment acts to change the polarity of materials such as the hydrophilization of metals and polymers [33, 34]. Although plants are living things, the polarity of their surfaces is likely to be altered by plasma treatment. Because membrane polarity affects endocytosis [35], proteins may be transferred into tobacco, Arabidopsis and rice cells through endocytosis stimulated by altered membrane polarity. The mechanism of how proteins are introduced into plant cells will be elucidated in future studies.

## Supporting information

S1 Table Primers and single-strand DNA sequences used in this study. (XLSX)
